# Gene mutation analysis using next‐generation sequencing and its clinical significance in patients with myeloid neoplasm: A multi‐center study from China

**DOI:** 10.1002/cam4.5690

**Published:** 2023-02-17

**Authors:** Junnan Li, Li Pei, Simin Liang, Shuangnian Xu, Yi Wang, Xiao Wang, Yi Liao, Qian Zhan, Wei Cheng, Zesong Yang, Xiaoqiong Tang, Hongbin Zhang, Qing Xiao, Jianbin Chen, Lin Liu, Li Wang

**Affiliations:** ^1^ Department of Hematology The First Affiliated Hospital of Chongqing Medical University Chongqing People's Republic of China; ^2^ Department of Hematology The First Affiliated Hospital of Army Medical University(Southwest Hospital) Chongqing China; ^3^ Department of Hematology Shaanxi Provincial People's Hospital Xi'An Shaanxi China; ^4^ Department of Oncology and Hematology Chongqing University Affiliated Center Hospital Chongqing China

**Keywords:** acute myeloid leukemia, gene mutation, myeloid neoplasm, next‐generation sequencing

## Abstract

**Background:**

Myeloid neoplasms (MN) tend to relapse and deteriorate. Exploring the genomic mutation landscape of MN using next‐generation sequencing (NGS) is a great measure to clarify the mechanism of oncogenesis and progression of MN.

**Methods:**

This multicenter retrospective study investigated 303 patients with MN using NGS from 2019 to 2021. The characteristics of the mutation landscape in the MN subgroups and the clinical value of gene variants were analyzed.

**Results:**

At least one mutation was detected in 88.11% of the patients (267/303). *TET2* was the most common mutation in the cohort, followed by *GATA2, ASXL1, FLT3, DNMT3A*, and *TP53*. Among patients with myeloid leukemia (ML), multivariate analysis showed that patients aged ≥60 years had lower overall survival (OS, *p* = 0.004). Further analysis showed *TET2, NPM1, SRSF2*, and *IDH1* gene mutations, and epigenetic genes (*p* < 0.050) presented significantly higher frequency in older patients. In patients with myelodysplastic syndrome (MDS) and myelodysplastic neoplasms (MPN), univariate analysis showed that *BCORL1* had a significant impact on OS (*p* = 0.040); however, in multivariate analysis, there were no factors significantly associated with OS. Differential analysis of genetic mutations showed *FLT3, TP53, MUC16, SRSF2*, and *KDM5A* mutated more frequently (*p* < 0.050) in secondary acute myeloid leukemia (s‐AML) than in MDS and MPN. *TP53, U2AF1, SRSF2*, and *KDM5A* were mutated more frequently (*p* < 0.050) in s‐AML than in primary AML. *KDM5A* was observed to be restricted to patients with s‐AML in this study, and only co‐occurred with *MUC16* and *TP53* (2/2, 100%). Another mutation was *MUC16*, and its co‐occurrence pattern differed between s‐AML and AML. MUC16 mutations co‐occurred with *KDM5A* and *TP53* in 66.7% (2/3) of patients with s‐AML and co‐occurred with *CEBPA* in 100% (4/4) of patients with AML.

**Conclusions:**

Our results demonstrate different genomic mutation patterns in the MN subgroups and highlight the clinical value of genetic variants.

## Graphical abstract

A multi‐center retrospective study, investigated patients of MN based on next‐generation sequencing (NGS).

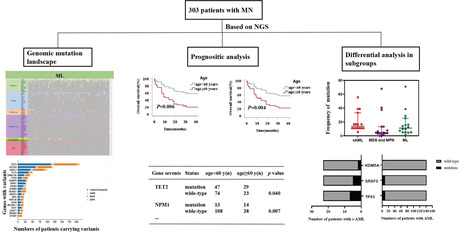

## 1 BACKGROUND

Myeloid neoplasms (MN) are clonal disorders of hematopoietic stem cells, including myeloid leukemia (ML), myelodysplastic syndrome (MDS), myelodysplastic neoplasms (MPN), and myelodysplastic syndrome/myeloid leukemia (MDS/MPN), which mainly include acute myeloid leukemia (AML) and chronic myeloid leukemia (CML).^1^ Despite being derived from homologous myeloid progenitors, this group of diseases represents a highly heterogeneous state in cytogenetic and molecular alterations and shares a tendency of progression to high malignancy of AML, which is known as secondary acute myeloid leukemia (s‐AML), and is associated with poor prognosis and relapses.^2^

Detecting genetic alterations in MN is a routine practice for accurate diagnosis and targeted therapeutic approaches.^2^, ^3^, ^4^ Traditional testing methods, including Sanger sequencing, real‐time polymerase chain reaction (PCR), reverse transcription (RT)‐PCR, chromosome karyotype analysis, and fluorescence in situ hybridization (FISH),^5^ provide appropriate molecular results but require large amounts of nucleic acids to evaluate individual genes.^6^ With its considerable sensitivity and measurement capability, next‐generation sequencing (NGS) has gradually become a validated tool for detecting genetic variants.^7^ Recent advances in NGS have revealed that *SF3B1, ASXL1*, and *TP53* mutations are helpful in the diagnosis of MDS.^8^ *TP53* and *ASXL1* mutations are associated with poor prognosis and a high risk of s‐AML transformation.^9^ *FLT3, JAK2*, and *IDH* mutations have been used in the choice of targeted drugs for AML.^10^ Moreover, NGS is a powerful tool that has been applied in micro residual disease testing of MN.^8^, ^11^, ^12^

Despite the varied knowledge of the molecular genetics of hematological neoplasms, the prognostic relevance and clinical value of genetics are not entirely consistent with heterogeneity and complexity. Thus, we undertook a multi‐center research and applied NGS to explore the characteristics of the genomic mutation landscape in subgroups of MN and clarify the prognostic relevance and value of these gene variants.

## 2 MATERIALS AND METHODS

### 2.1 Patients and samples

This study was conducted by the Department of Hematology of the First Affiliated Hospital of Chongqing Medical University, in cooperation with other hematology centers in the southwest region of China. All patients were recently diagnosed with MN at the study centers between 2019 and 2021, including ML, MDS, MPN, and MDS/MPN. According to the WHO classification, the diagnosis and classification were based on multidisciplinary approaches; s‐AML was defined as AML with antecedent hematological disease. Cytogenetic risk stratification was based on the revised 2017 European Leukemia Net risk stratification.^13^ Finally, 303 patients with MN were enrolled in the study, and patient characteristics and clinical outcomes were recorded, including age, sex, blood routine test results, chromosomal karyotypes, and treatments. All samples (peripheral blood or bone marrow) from patients underwent NGS analysis with informed consent. The study protocol was approved by the ethics committee of the study centers (2021‐342).

### 2.2 Targeted sequencing and analysis

#### 2.2.1 Panels selection

The gene panels used in this study were chosen based on prognostic and diagnostic significance and covered nine major functional categories that indicate the important genetic events in MN pathogenesis.^14^ In this study, we screened a 130‐gene commercial panel of recurrent gene variants in hematologic malignancies with potential significance in MN, including DNA methylation‐associated genes, transcription factors genes, spliceosome‐complex genes, activated signaling genes, cohesin‐complex genes, chromatin modifier genes, regulation of cell cycle, differentiation, and proliferation genes, tumor suppressor genes, and apoptosis genes (Table 1).

**TABLE 1:** The 130 captured purpose genes according to functional classification.

Functional Cluster	Genes
DNA methylation‐associated genes	DNMT3A, TET2, KDM5A, DOT1L, KMT2A, KMT2C, KMT2D
Transcription factors genes	RUNX1, CEBPA, EP300, CREBBP, IDH1, IDH2, IKZF1, MYC, ETV6, NCOR1, CUX1, GATA1, WT1, GATA2, RB1, MECOM, PML, NCOR2, CRLF2, IL7R, NFE2, PAX5, SRP72
Spliceosome‐complex genes	DKC1, SRSF2, SF3B1, U2AF1, ZRSR2, SF1, PRPF40B, SF3A1, PRPF8, U2AF2, ZFP36L1
Activated signaling genes	FLT3, HRAS, NRAS, KRAS, CBL, KIT, NF1, PTPN11, ABL1, ROBO1, MPL, PDGFRA, ROBO2, STAT3, SETBP1, JAK1, MYD88, CSF3R, JAK2, JAK3, FBXW7, CALR, NOTCH1, NOTCH2, CCND1, GNAS, PTEN, TYK2, SMARCA2, ZAP70
Cohesin‐complex genes	RAD21, SMC1A, SMC3, STAG1, STAG2, MUC16, CTCF
Chromatin modifier genes	ASXL1, ASXL2, EZH2, BCOR, PHF6, ARID1A, ARID1B, BCORL1, KDM6A, ATRX
Cell cycle, differentiation, and proliferation regulation genes	ATG2B, CDKN2A, COL12A1, FAT1, GFI1, RARA, BRAF, SH2B3/LNK, PTPRT, DDX11, NPM1, NT5C2, PPM1D, SUZ12, TERT, SMN1
Tumor suppressor genes	ATM, BLM, TP53, BRCA1, BRCA2
Apoptosis genes	HAX1, TERC
Others	AKNRD26, PBRM1, EPPK1, PIGA, CROCC, TTN, CSMD1, WAC, DDX41, CCND2, DIS3, ZMYM3, BRINP3, CELA2A, GSKIP, SBDS, ZNF608, ETNK1

#### 2.2.2 DNA extraction and identification

Bone marrow or peripheral blood (2 mL) were collected in an EDTA tube, and centrifugal separation was performed with erythrocyte lysates to obtain mononuclear cells. This was followed by DNA extraction using the Blood Genomic DNA extraction kit (0.1–1 mL; Beijing Tian Yuan Biotech Co., Ltd.). DNA concentration of the samples was quantified using a Qubit fluorometer (Thermo Fisher Scientific).

#### 2.2.3 Library preparation and analysis

Library preparation was performed by amplification and capture using the NGS gene panel detection library construction kit (Shanghai yuanqi Bio‐pharmaceutical Technology Co., Ltd.) and PE150 sequencing on Nextseq 550 Sequencing System (Illumina). The primary data were aligned to the human reference genome at NCBI, Clinvar, dbSNP (V138), COSMIC, and Human Genome database (HG19) with the determination of point mutations (SNV), insertions and deletions (INDEL), and pathogenic mutations.

### 2.3 Statistical analyses

The endpoint was defined as the date of death or the last follow‐up date, and overall survival (OS) was measured from the time of initial diagnosis to the endpoint. All statistical procedures were performed using the software packages SPSS (version 26.0; IBM Corporation) and GraphPad Prism (Datamatics 8.0). A non‐parametric test (Mann–Whitney *U* test) was used to compare the frequency of mutation among different diseases, and the COX proportional hazards regression model was used to identify the prognostic value of genetic variants. *χ* ^2^ test and Fisher's exact test were used for categorical variables. *p*‐values <0.05 were considered statistically significant.

## 3 RESULTS

### 3.1 Patient cohort and clinical characteristics

**FIGURE 1:**
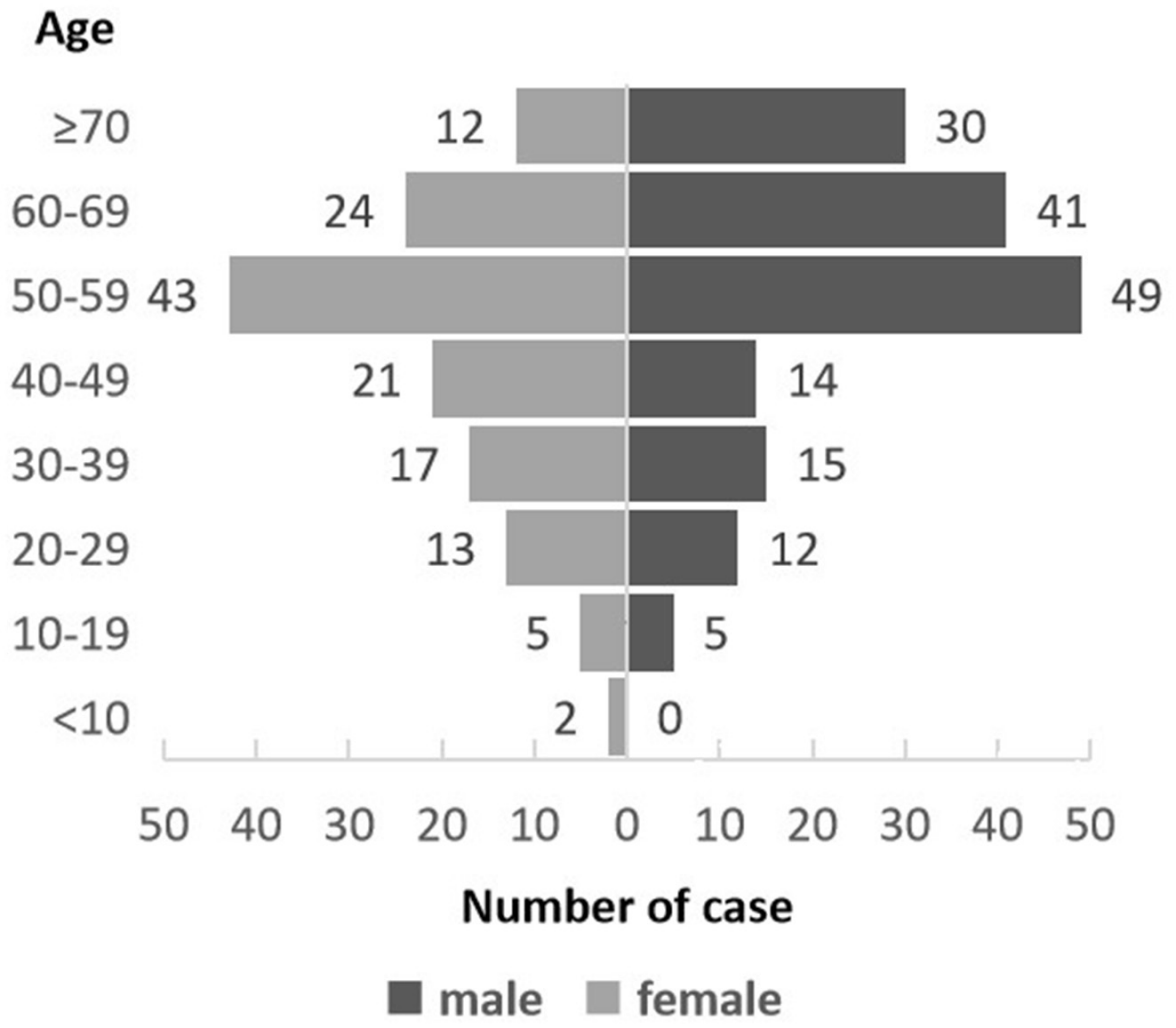
Age structure diagram of patients with myeloid neoplasms (MN).

A total of 303 patients with MN were retrospectively enrolled in the study, and their clinical characteristics are summarized in Table 2. The cohort included 173 patients with ML, 118 with MDS, two with MPN, and 10 with MDS/MPN. Within ML, 165 patients were diagnosed with AML (23 patients were defined as having s‐AML with antecedent hematological disorders of MDS or MPN, and 142 patients were defined as having de novo AML). Another eight patients had other types of ML (four patients with MAL, two with BAL, and two with CML). The median age of the patients was 55 years (range 6–86 years), with 54.78% (166/303) males and 45.22% (137/303) females. The highest proportion of patients aged 50–59 years was 30.36% (92/303), and this proportion gradually decreased in the two age groups. (Figure 1).

**TABLE 2:** Clinical characteristics of 303 patients of MN.

Clinical variables	MN median (range; count%)	ML	MDS	MPN and MDS/MPN
Total number (number)	303	173	118	12
Sex (female/male)	137/166	83/90	51/67	3/9
Median age (years old)	55 (6–86)	54 (9–86)	56.50 (6–86)	59.50 (50–75)
Median WBC count (×10^9^/L)	3.46 (0.69–682.70)	9.34 (0.69–682.7)	2.29 (0.70–32.82)	19.32 (3.16–253.43)
Median hemoglobin (g/L)	77 (17–165)	78 (28–147)	74 (16.40–165)	71.50 (48–106)
Median platelets (×10^9^/L)	45 (2–4384)	43 (2–4384)	46.50 (3–405)	121.50 (22–598)
Myeloid neoplasm subtype				
AML		165		
s‐AML		23		
de novo AML		142		
MAL		4		
BAL		2		
CML		2		
MDS			118	
MDS‐MLD			34	
MDS‐SLD			5	
MDS‐RS‐MLD			6	
MDS‐5q‐			3	
MDS‐EB1			34	
MDS‐EB2			22	
MDS‐U			14	
MPN				2
CMML				2
MPN/MDS				8
Chromosomal karyotypes				
Good	56	28	27	1
Intermediate	180	95	75	10
Poor	67	50	16	1
Number of mutation				
0	36	14	21	1
1	49	29	19	1
2	50	30	19	1
≥3	167	100	59	8
Treatments				
Chemotherapy and demethylation therapy	180	136	38	6
HSCT	25	20	5	0
Supportive treatment	98	17	75	6
Median follow‐up term (months)	3 (0–197)	3 (0–197)	2.5 (0.1–80)	1.75 (0.2–81)

Abbreviations: AML, acute myeloid leukemia; BAL, Biphenotype cellular leukemia; CML, Chronic myeloid leukemia; CMML, chronic myelomonocytic leukemia; HSCT, hematopoietic stem cell transplantation; MAL, Mixed cellular leukemia; MDS, myelodysplastic syndrome; MDS‐5q‐, myelodysplastic syndrome with isolated del(5q); MDS‐EB1, myelodysplastic syndrome with excess blasts one; MDS‐EB2, myelodysplastic syndrome with excess blasts two; MDS‐MLD, myelodysplastic syndrome with single lineage dysplasia; MDS‐RS‐MLD, myelodysplastic syndrome with sideroblasts and multilineage dysplasia; MDS‐SLD, myelodysplastic syndrome with multilineage dysplasia; MDS‐U, myelodysplastic syndrome unclassifiable; MN, myeloid neoplasm; MPN, myeloproliferative neoplasm; WBC, white cell count.

According to chromosomal karyotype classification,^15^ 18.48% (56/303), 59.41% (180/303), and 22.11% (67/303) of the patients were classified into the good prognosis group, intermediate group, and poor prognosis group, respectively (Table 2). All patients underwent NGS analysis; 296 test samples were bone marrow samples and seven samples were peripheral blood samples. At least one mutated gene was identified in 88.11% (267/303) of the patients, and complex variations (more than three gene mutations) were detected in 62.54% (167/303).

The treatments included symptomatic support therapy, chemotherapy, demethylation therapy, and stem cell transplantation (autologous hematopoietic stem cell transplantation (HSCT) and allogeneic HSCT). Approximately 59.41% (180/303) of the patients received chemotherapy and demethylation therapy, 8.25% (25/303) received stem cell transplantation, and 32.34% (98/303) received supportive therapy with blood transfusion and anti‐infection treatment. All patients completed the follow‐up plans with a median follow‐up time of 3 months (Table 2).

### 3.2 Gene mutation landscape in the MN subgroups

**FIGURE 2:**
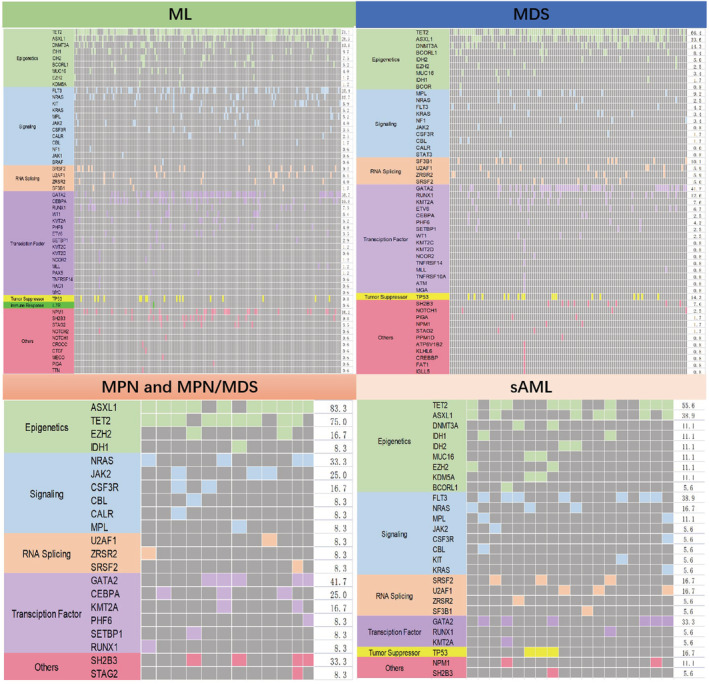
Variant landscape of diagnostic samples of 303 patients and subgroups of ML, MDS, MPN, and s‐AML. Each column represents a patient. Colors represent the type of gene mutation. The percentage of patients with mutations in each gene is presented on the right line. MDS/MPN, myelodysplastic syndrome/myeloproliferative neoplasm; MDS, myelodysplastic syndrome; ML, myeloid leukemia; MPN, myeloproliferative neoplasm; S‐AML, secondary acute myeloid leukemia.

**FIGURE 3:**
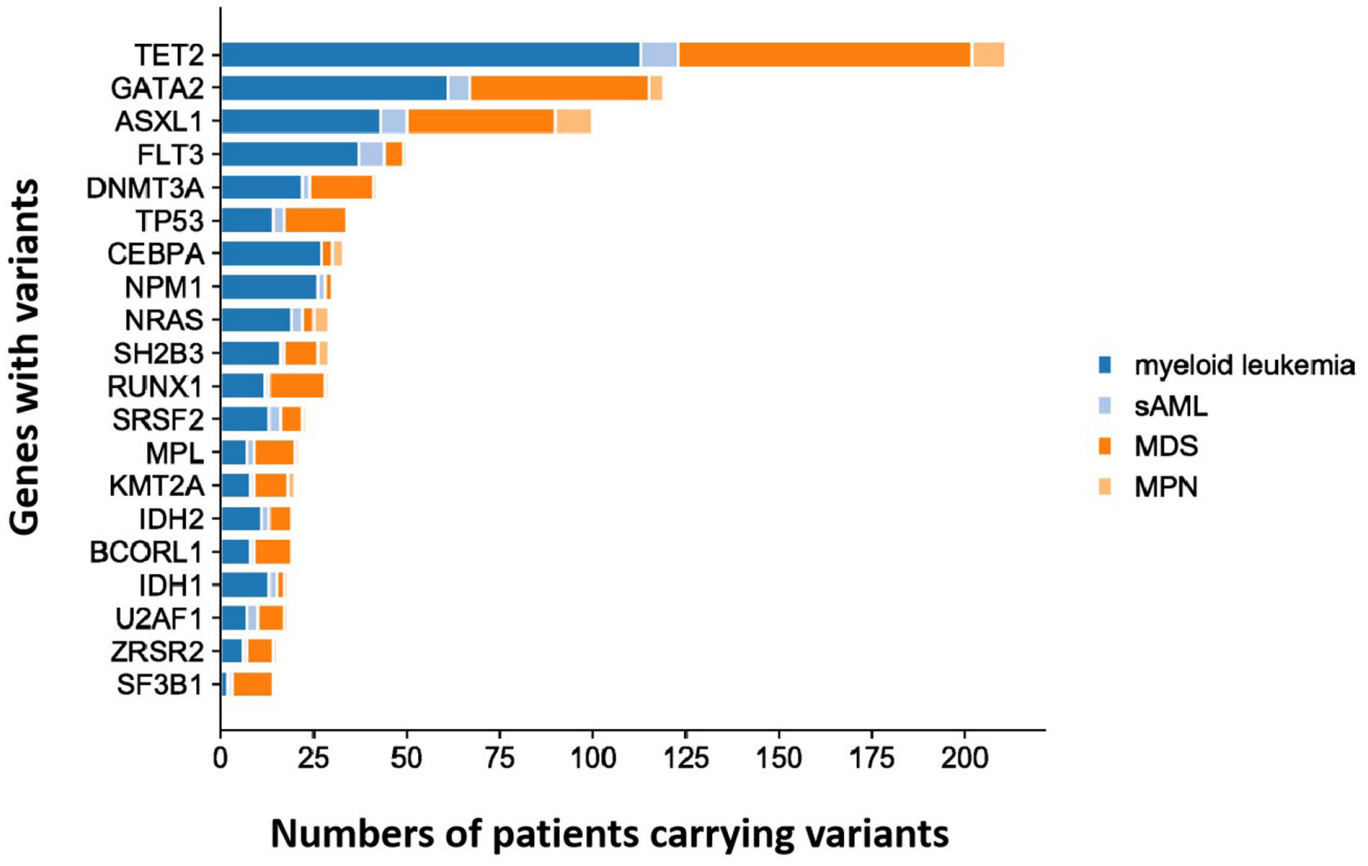
Number of patients carrying variants in top 20 high‐frequency mutations of our study. Dfferent color represents different disease type of MN. MDS/MPN, myelodysplastic syndrome/myeloproliferative neoplasm; MDS, myelodysplastic syndrome; MPN, myeloproliferative neoplasm; s‐AML, secondary acute myeloid leukemia.

The distribution of mutations in the subgroups is depicted in the mutation landscape (Figure 2). As expected, the three common variants in the ML cohort were *TET2* (71.1%), *GATA2* (38.7%), and *ASXL1* (28.9%); in MDS, *TET2* (66.4%), *GATA2* (41.2%), and *ASXL1* (33.6%); and in MPN and MPN/MDS, *ASXL1* (83.3%), *TET2* (75%), *NRAS* (33.3%), and *SH2B3* (33.3%). The most common variants in patients with s‐AML were *TET2* (55.6%), *FLT3* (38.9%), *ASXL1* (38.9%), and *GATA2* (33.3%). The top 20 common gene variants were *TET2, GATA2, ASXL1, FLT3, DNMT3A, TP53, CEBPA, NPM1, NRAS, SH2B3, RUNX1, SR2B3, RUNX1, SRSF2, MPL, KMT2A, IDH2, BCORL1, IDH1, U2AF1, ZRSR2*, and *SF3B1*, while other genes exhibited variation at extremely low frequency. The mutation status in the different subgroups is shown as bar graphs (Figure 3).

#### 3.2.1 Mutational co‐occurrence and mutual exclusion patterns

**FIGURE 4:**
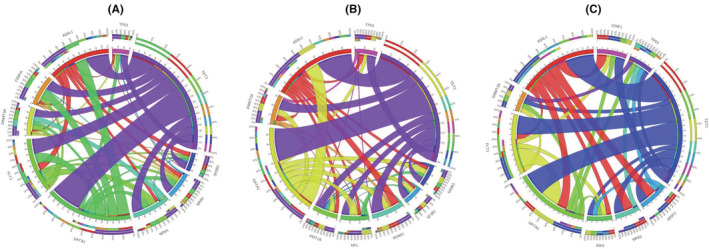
Mutational co‐occurrence and mutual exclusion in subgroups of MN. The length of the arc represents the relative frequencies of the first gene mutations, and the width of the ribbon depicts the relative frequencies of co‐occurrences. (A) Mutational co‐occurrence and mutual exclusion in ML patients. (B) Mutational co‐occurrence and mutual exclusion in patients of MDS, MPN, and MDS/MPN. (C) Mutational co‐occurrence and mutual exclusion in s‐AML patients.

Co‐occurrence and mutual exclusion of high‐frequency variants in the subgroups were analyzed. In patients with ML, significant co‐occurrence was detected between *TET2* mutations and *GATA2, FLT3, ASXL1, NPM1*, and *CEBPA* mutations. *GATA2* with *FLT3, NPM1*, and *CEBPA* mutations regularly occurred. Regarding the mutual exclusion of gene variants, the data from *TP53* was a standout; we could not identify co‐occurrence with *NPM1, FLT3*, or *CEBPA* mutations and low frequency with other gene variants (Figure 4A).

In MDS, MPN, and MDS/MPN, *TET2* significantly co‐occurred with *GATA2* and *ASXL1* variants. *GATA2* and *ASXL1* also co‐occurred. In the mutual exclusion pattern, *TP53* had low frequency compared with other gene variants and had no co‐occurrence with *SH2B3, SF2B1*, and *MPL* (Figure 4B).

In s‐AML, the co‐occurrence of *TET2* with *GATA2* and *ASXL1* mutations was significant, and *GATA2* and *ASXL1* also co‐occurred. In contrast, mutual exclusivity was observed between *TP53* and *FLT3, FLT3* and *ASXL1, ASXL1* and *DNMT3*, and *FLT3* and *SRSF2* (Figure 4C).

### 3.3 Prognostic analysis

The mean OS of patients with ML was 9.36 ± 1.42 months, while the OS of MDS and MPN patients was 9.06 ± 1.42 months, with no significant difference (*p* = 0.617). The relevance between prognosis, clinical characteristics, and gene variants was evaluated. In univariate analysis, the prognostic factors with *p* < 0.200 were considered essential and were enrolled in multivariate analysis. Patients with ML, s‐AML, MDS, and MPN (including MDS/MPN) were analyzed.

#### 3.3.1 Prognostic analysis of ML

**FIGURE 5:**
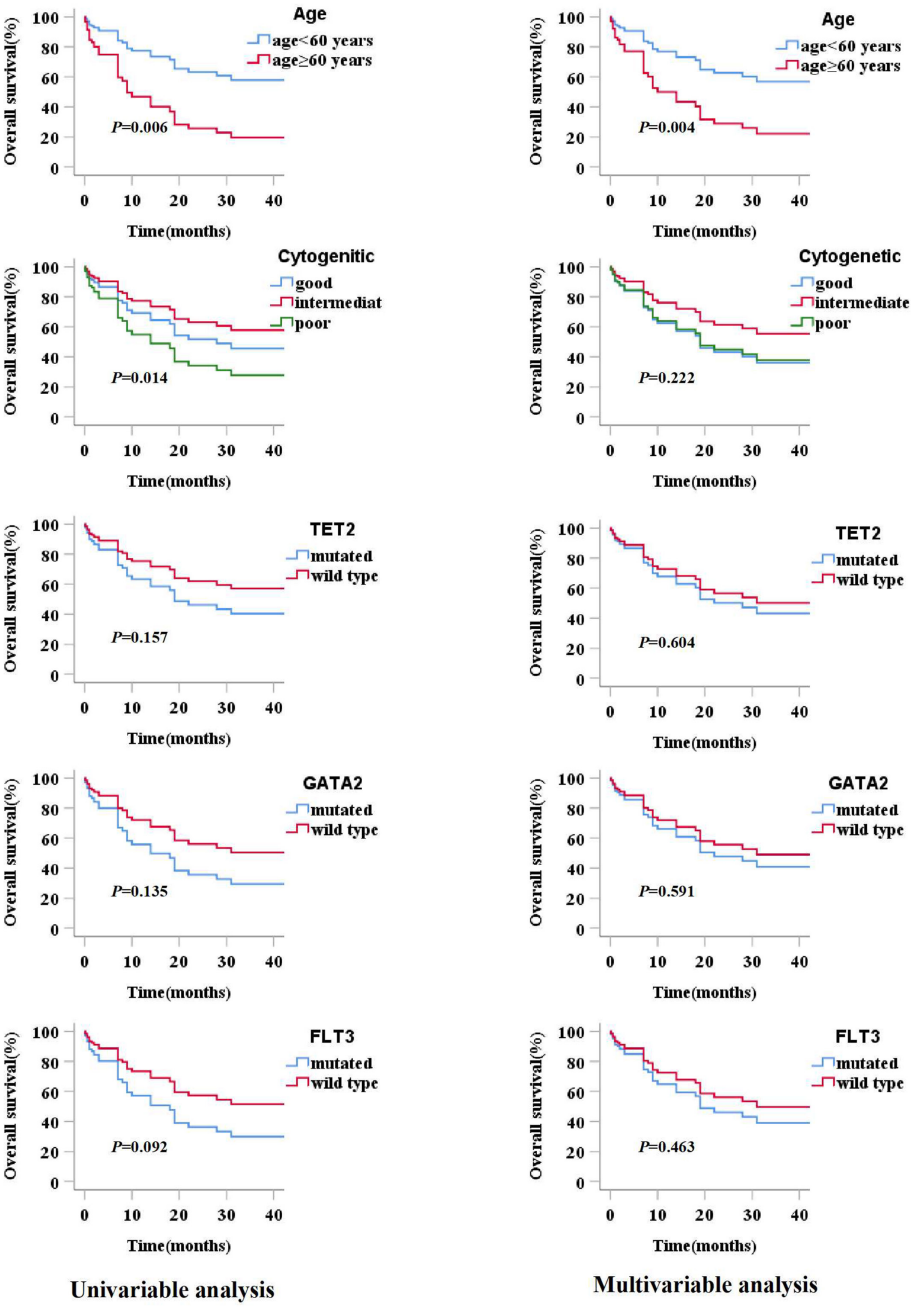
Prognostic analysis of myeloid leukemia(ML) cohort according to clinical characteristics and gene mutations. The factors of age, cytogenetic, *TET2, GATA2*, and *FLT3* mutation had enrolled in multivariate analysis. Univariate analysis showed Patients in age ≥ 60 years group had a significantly inferior overall survival (OS) compared with patients in age < 60 years group (*p* = 0.006), the intermediate and poor cytogenetic groups showed significant difference in OS (*p* = 0.014), but multivariate analysis indicated only factor of age was an independent prognostic factor of ML.

Age, WBC count, chromosomal karyotypes, stem cell transplantation, and gene variants were included for survival analysis. Univariate analysis showed older patients (age ≥ 60 years) and complex karyotypes had significant negative impacts on the OS of patients with ML (*p* = 0.006). *TET2*, *GATA2*, and *FLT3* mutations were considered as other essential factors (*p* < 0.200) and were included in the multivariate analysis. In the multivariate analysis, only age (≥60 years) was observed to have a significant unfavorable impact on OS (*p* = 0.004), and older patients with ML demonstrated lower OS (Table 3). Survival curves for all factors included in the multivariate analysis are shown in Figure 5.

**TABLE 3:** Prognostic implications according to clinical characteristics and detected gene mutations by univariate analysis and multivariate analysis and their *p* values for ML.

Variables	OS(ML)			
	Univariate analysis HR(CI 95%)	*p* value	Multivariate analysis HR(CI 95%)	*p* value
Age ≥ 60 years	2.519 (1.302–4.873)	0.006*	0.375 (0.192–0.734)	0.004*
WBC≥100 × 10^9^/L	1.805 (0.545–5.979)	0.334		
Chromosomal karyotypes				
Good versus poor	0.618 (0.224–1.701)	0.343	1.046 (0.342–3.199)	0.937
moderate versus poor	0.430 (0.218–0.847)	0.014*	0.609 (0.274–1.350)	0.222
Stem cell transplants(yes versus no)	0.837 (0.324–2.165)	0.714		
Gene mutation(yes versus no)	1.472 (0.348–6.228)	0.599		
TET2 mutated versus wt	1.612 (0.832–3.122)	0.157	1.215 (0.582–2.535)	0.604
GATA2 mutated versus wt	1.784 (0.836–3.811)	0.135	1.256 (0.547–2.885)	0.591
ASXL1 mutated versus wt	1.469 (0.767–2.815)	0.246		
FLT3 mutated versus wt	1.819 (0.908–3.646)	0.092	1.343 (0.611–2.955)	0.463
CEBPA mutated versus wt	0.785 (0.306–2.014)	0.614		
NPM1 mutated versus wt	1.182 (0.460–3.033)	0.729		
NRAS mutated versus wt	0.870 (0.308–2.455)	0.793		
DNMT3A mutated versus wt	0.885 (0.313–2.502)	0.817		
SH2B3 mutated versus wt	0.667 (0.087–5.140)	0.698		
TP53 mutated versus wt	1.575 (0.554–4.482)	0.394		

*Note*: COX proportional hazard regression analysis was used for evaluations of each variables.

Abbreviations: ML, myeloid leukemia; OS, overall survival; WBC, white blood cell; Wt, wide‐type.

* Statistical difference (*p* < 0.05) was observed.

To further explore the inferior prognosis in older patients with ML, genetic mutation events between the younger and older patient groups were compared. High‐frequency mutated genes and functional classifications were also included. *TET2* (55.77% vs. 38.84%, *p* = 0.040), *NPM1* (26.92% vs. 10.74% *p* = 0.007), *SRSF2* (19.23% vs. 3.31% *p* = 0.000), *IDH1* (19.23% vs. 2.48% *p* = 0.000), and epigenetic genes (82.69% vs. 66.12%, *p* = 0.000) presented significantly higher frequency in older (≥60 years) patients (Table 4).

**TABLE 4:** Genetic abnormalities of ML patients stratified by age groups.

Genes events	Status	Patients of age < 60 years (*n*)	Patients of age ≥ 60 years (*n*)	*χ* ^2^	*p* value
TET2	Mutation	47	29		
	Wide‐type	74	23	4.23	0.040*
GATA2	Mutation	30	13		
	Wide‐type	91	39	0.001	0.977
ASXL1	Mutation	24	17		
	Wide‐type	97	35	3.325	0.068
FLT3	Mutation	28	15		
	Wide‐type	93	37	0.634	0.426
CEBPA	Mutation	21	7		
	Wide‐type	100	45	0.407	0.524
NPM1	Mutation	13	14		
	Wide‐type	108	38	7.228	0.007*
NRAS	Mutation	15	6		
	Wide‐type	106	46	0.025	0.874
DNMT3A	Mutation	14	9		
	Wide‐type	107	43	1.039	0.308
SH2B3	Mutation	6	1		
	Wide‐type	115	51	0.863	0.353
TP53	Mutation	11	6		
	Wide‐type	110	46	0.246	0.620
SRSF2	Mutation	4	10		
	Wide‐type	117	42	12.401	0.000*
IDH1	Mutation	3	10		
	Wide‐type	118	42	14.685	0.000*
Epigenetic genes	Mutation	80	43		
	Wide‐type	41	9	4.864	0.027*
Transcription factors genes	Mutation	62	19		
	Wide‐type	59	33	3.157	0.076
Activated signaling genes	Mutation	56	26		
	Wide‐type	65	26	0.202	0.653

* Statistical difference (*p* < 0.05) was observed.

#### 3.3.2 Prognostic analysis of MDS, MPN, and MDS/MPN

**FIGURE 6:**
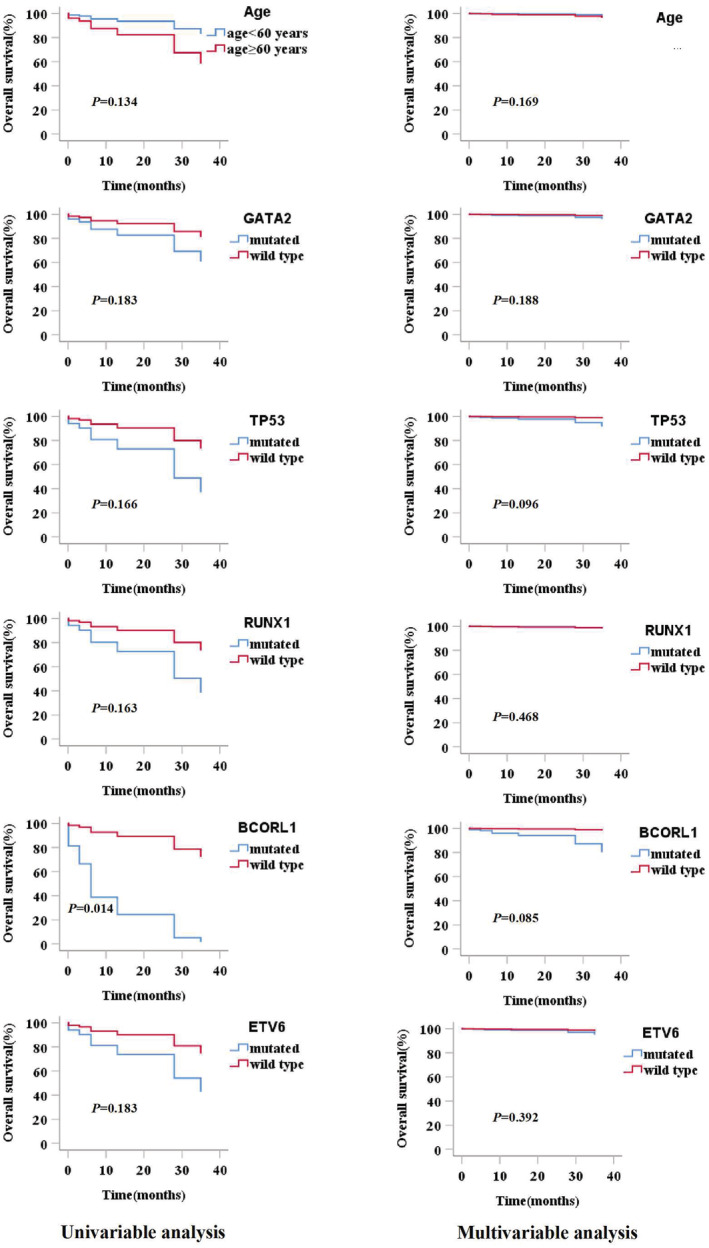
Prognostic analysis of MDS, MPN, and MDS/MPN cohort according to clinical characteristics and gene mutations. The factors of age and *GATA2, TP53, RUNX1, BCORL1, ETV6* mutation had enrolled in multivariate analysis. Univariate analysis showed patients with *BCORL1* mutation had a significantly inferior OS (*p* = 0.014), but multivariate analysis indicated the mutation was not an independent prognostic factor of MDS and MPN (*p* = 0.085).

Clinical prognostic factors included age, WBC count, chromosomal karyotypes, treatments, gene variants, and single mutated genes. In univariate analysis, patients with *BCORL1* mutations showed a significantly reduced OS (*p* = 0.040); however, other factors showed no significant impact on survival. According to the *p* value in the univariate analysis, age and *GATA2, TP53, RUNX1*, and *ETV6* mutations were considered in the multivariate analysis (Table 5). In multivariate analysis, all factors showed *p* ≥ 0.050, which indicated that clinical features and gene variants were not effective prognostic factors of MDS and MPN in the study. The survival curves for all factors included in the multivariate analysis are shown in Figure 6.

**TABLE 5:** The variables in univariate analysis and multivariate analysis of survival and their *p* values for MDS and MPN.

Variables	OS(MDS and MPN)			
	Univariate analysis HR (CI 95%)	*p* value	Multivariate analysis HR (CI 95%)	*p* value
Age ≥ 60 years	0.355 (0.092–1.376)	0.134	0.366 (0.088–1.532)	0.169
WBC≥100 × 109/L	22.843 (0–4.670E+23)	0.671		
Chromosomal karyotypes				
Good versus poor	0 (0–3.151 E+230)	0.962		
Moderate versus poor	5.810 (0.119–2.840)	0.501		
Demethylation therapy versus supportive treatment	2.115 (0.435–10.294)	0.353		
Gene mutation(yes versus no)	0.914 (0.113–7.367)	0.933		
TET2 mutated and wt	1.052 (0.292–3.782)	0.939		
GATA2 mutated and wt	2.394 (0.663–8.649)	0.183	2.437 (0.646–9.188)	0.188
ASXL1 mutated and wt	0.630 (0.133–2.983)	0.561		
DNMT3A mutated and wt	1.843 (0.384–8.842)	0.443		
TP53 mutated and wt	3.199 (0.617–16.585)	0.166	4.491 (0.766–26.342)	0.096
RUNX1 mutated and wt	3.103 (0.634–15.195)	0.163	2.023 (0.302–13.559)	0.468
SF3B1 mutated and wt	0.044 (0–1509.585)	0.558		
MPL mutated and wt	0.929 (0.115–7.498)	0.945		
SH2B3 mutated and wt	1.485 (0.314–7.032)	0.618		
BCORL1 mutated and wt	12.400 (1.124–136.750)	0.040*	12.317 (0.862–186.072)	0.085
KMT2A mutated and wt	0.046 (0–3.187E+06)	0.730		
ETV6	2.886 (0.606–13.743)	0.183	2.674 (0.282–25.399)	0.392

*Note*: COX proportional hazard regression analysis was used for evaluations of each variables.

Abbreviations: MDS, myelodysplastic syndrome; MPN, myeloproliferative neoplasm; OS, overall survival; WBC, white blood cell.

* Statistical difference (*p* < 0.05) was observed.

#### 3.3.3 Prognostic analysis of s‐AML

Patients with s‐AML were the smallest subgroup. Additionally, we performed further analysis for potential prognostic factors, especially for the high frequency of gene variants. However, in the univariate analysis, survival was not significantly different based on factors such as age, WBC count, and gene variants (Table 6).

**TABLE 6:** The variables in univariate analysis of survival and their *p* values for s‐AML.

Variables	OS(s‐AML)	
	Univariate analysis HR(CI 95%)	*p* value
Age ≥ 60 years	0.775 (0.242–2.481)	0.667
WBC≥10 × 10^9^/L	0.696 (0.223–2.178)	0.534
TET2 mutated and wt	0.703 (0.210–2.353)	0.568
GATA2 mutated and wt	2.059 (0.395–10.729)	0.391
ASXL1 mutated and wt	0.799 (0.237–2.688)	0.716
FLT3 mutated and wt	1.490 (0.428–5.189)	0.531
NRAS mutated and wt	2.353 (0.471–11.762)	0.297
SRSF2 mutated and wt	1.827 (0.530–6.292)	0.340
U2AF1 mutated and wt	0.118 (0.014‐‐0.996)	0.050
TP53 mutated and wt	0.232 (0.036–2.234)	0.232

Abbreviations: OS, overall survival. WBC, white blood cell.

### 3.4 Differential analysis of gene variants among MN subgroups

#### 3.4.1 Comparison of mutational burden between the subgroups

**FIGURE 7:**
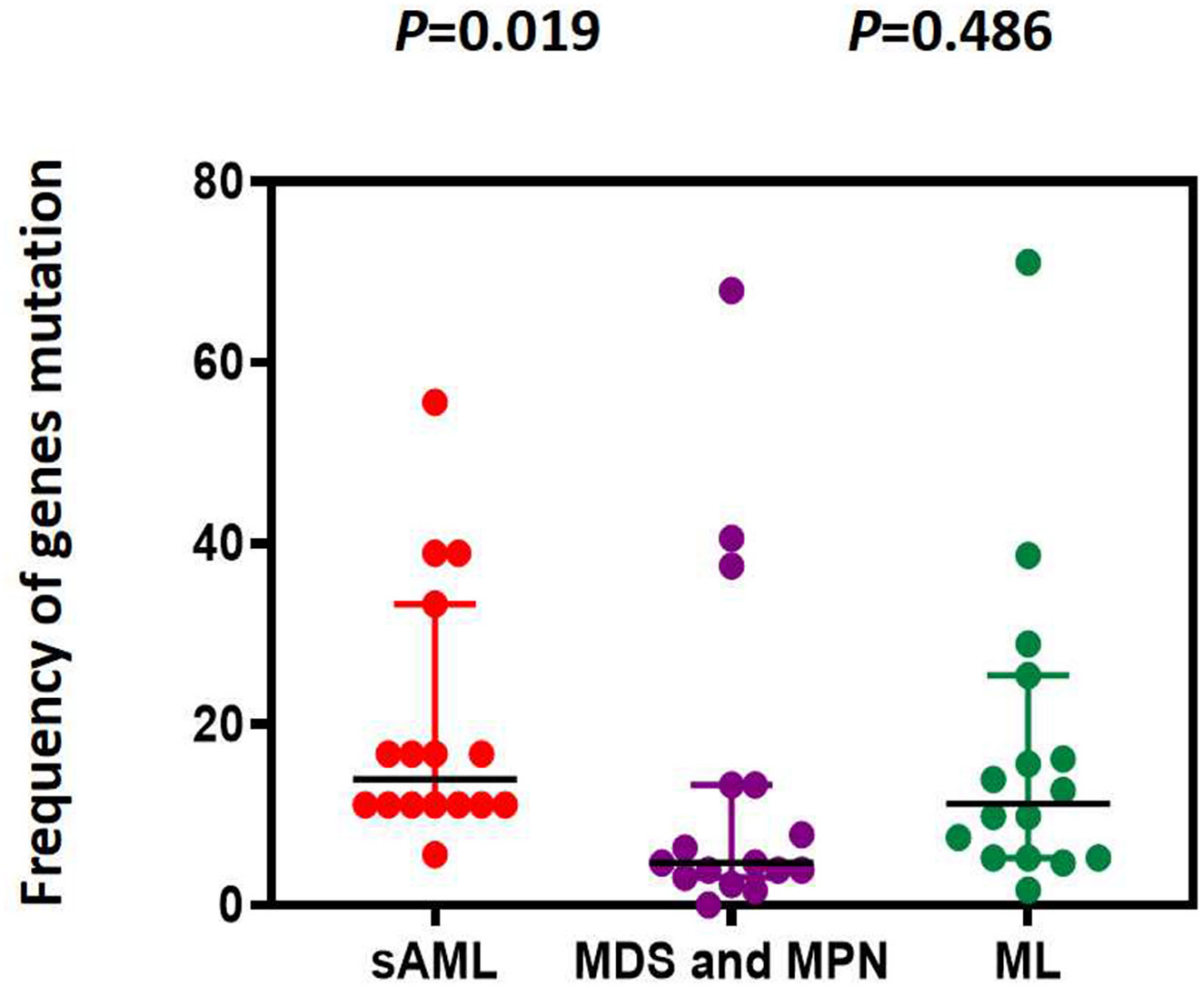
Comparison of gene mutations burden among subgroups of MN. The colors represents different disease, the dots represent number of patients with high frequency of mutation.

We selected and integrated the top 10 most common gene variants between ML, MDS, and MPN to evaluate the gene mutational burden; however, there was no significant difference (*p* = 0.486). In contrast, the mutational burden in s‐AML was higher than that in the MDS and MPN subgroups (*p* = 0.019; Figure 7).

**FIGURE 8:**
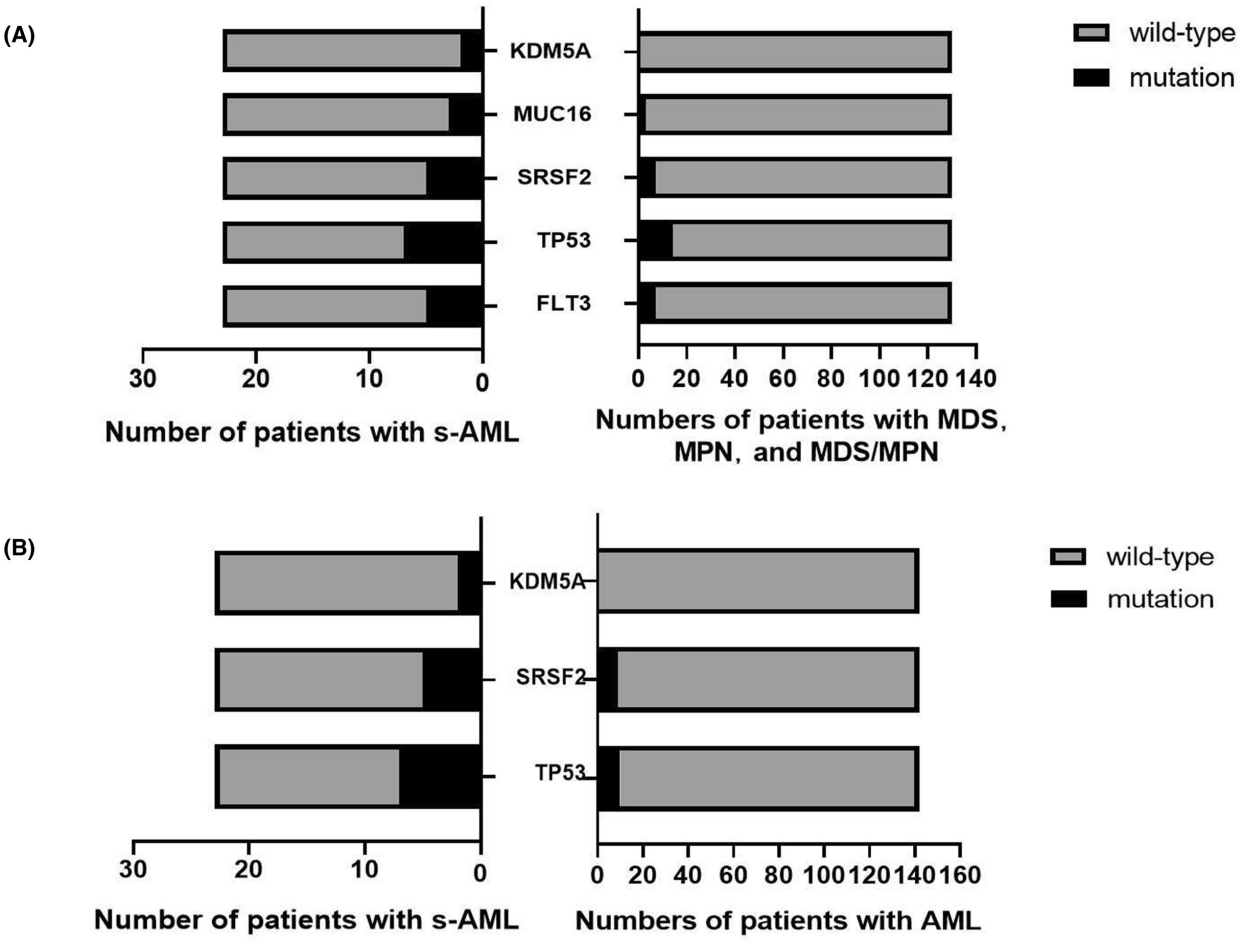
Differential analysis of genes status among subgroups of MN. (A) *FLT3, TP53, MUC16, SRSF2*, and *KDM5A* had significant higher mutated frequency in s‐AML than in MDS, MPN and MDS/MPN. (B) *TP53, U2AF1, SRSF2*, and *KDM5A* mutations had significant higher mutated frequency in s‐AML than AML. Color of bar diagram represents gene mutation status, height of the bar diagram represents number of patients.

To further understand the diversity of gene variants, we integrated high‐frequency gene variants into two subgroups for comparison. Thirteen variants were selected among s‐AML, MDS, and MPN. *FLT3* (*p* = 0.019), *TP53* (*p* = 0.020), *MUC16* (*p* = 0.044), *SRSF2* (*p* = 0.007), and *KDM5A* (*p* = 0.022) mutations were significantly more frequent in s‐AML (Table 7, Figure 8A). Simultaneously, 18 gene variants were screened and integrated between s‐AML and de novo AML; *TP53* (*p* = 0.003), *U2AF1* (*p* = 0.049), *SRSF2* (*p* = 0.029), and *KDM5A* (*p* = 0.019) mutations displayed higher frequencies in s‐AML (Table 8, Figure 8B).

**TABLE 7:** Differences of gene mutation status between s‐AML and MDS, MPN.

Genes	Status	Patients with MDS and MPN (*n*)	Patients with s‐AML (*n*)	*χ* ^2^	*p* value
TET2	Wild‐type	73	9		
	Mutation	57	14	2.277	0.131
GATA2	Wild‐type	72	19		
	Mutation	38	4	1.375	0.241
ASXL1	Wild‐type	91	16		
	Mutation	39	7	0.002	0.967
FLT3	Wild‐type	123	18		
	Mutation	7	5	5.146	0.023*
NRAS	Wild‐type	123	19		
	Mutation	7	5	2.614	0.106
TP53	Wild‐type	116	16		
	Mutation	14	7	4.830	0.028*
SRSF2	Wild‐type	123	18		
	Mutation	7	5	5.146	0.023*
U2AF1	Wild‐type	123	19		
	Mutation	7	4	2.614	0.106
NPM1	Wild‐type	128	22		
	Mutation	2	1		0.389
MUC16	Wild‐type	127	20		
	Mutation	3	3		0.044*
IDH2	Wild‐type	124	22		
	Mutation	6	1	0.000	1.000
EZH2	Wild‐type	126	21		
	Mutation	4	2		0.222
KDM5A	Wild‐type	130	21		
	Mutation	0	2		0.022*

* Statistical difference (*p* < 0.05) was observed.

**TABLE 8:** Differences of gene mutation status between s‐AML and AML, *TP53, U2AF1, SRSF2, KDM5A* mutations status showed significant difference between s‐AML and AML.

Genes	Status	Number of patients in AML (*n*)	Patients in s‐AML (*n*)	*χ* ^2^	*p* value
TET2	Wild‐type	81	9		
	Mutation	61	14	2.561	0.110
GATA2	Wild‐type	104	19		
	Mutation	38	4	0.916	0.339
ASXL1	Wild‐type	109	16		
	Mutation	33	7	0.558	0.455
FLT3	Wild‐type	107	18		
	Mutation	35	5	0.002	0.968
TP53	Wild‐type	132	16		
	Mutation	10	7	9.326	0.002*
U2AF1	Wild‐type	135	19		
	Mutation	7	4	3.140	0.076
SRSF2	Wild‐type	133	18		
	Mutation	9	5	4.226	0.040*
NRAS	Wild‐type	126	19		
	Mutation	16	4	0.241	0.624
DNMT3A	Wild‐type	124	19		
	Mutation	18	3	0.000	1.000
IDH2	Wild‐type	130	22		
	Mutation	12	1	0.068	0.795
EZH2	Wild‐type	141	21		
	Mutation	1	2		0.051
MUC16	Wild‐type	138	20		
	Mutation	4	3		0.057
NPM1	Wild‐type	116	22		
	Mutation	26	1	1.891	0.169
IDH1	Wild‐type	129	23		
	Mutation	13	0	1.198	0.274
KDM5A	Wild‐type	142	21		
	Mutation	0	2		0.019*
BCORL1	Wild‐type	134	23		
	Mutation	8	0	0.414	0.520
SH2B3	Wild‐type	135	23		
	Mutation	7	0		0.595
CEBPA	Wild‐type	115	22		
	Mutation	27	1	2.071	0.150

* Statistical difference (*p* < 0.05) was observed.

### 3.5 Two special differential genes in s‐AML

**FIGURE 9:**
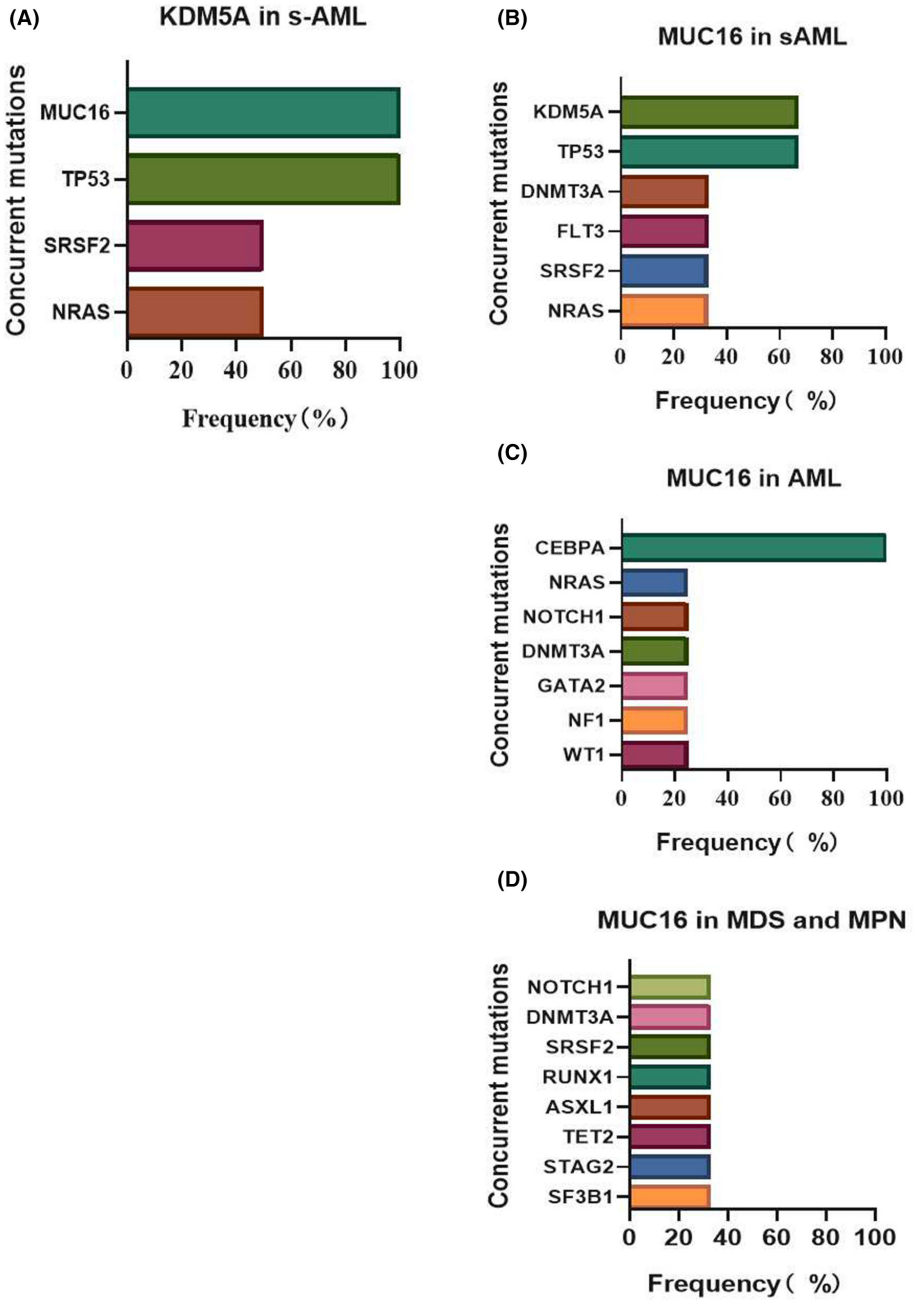
The concurrent mutations of special genes *KDM5A* and *MUC16*. Colors representing different mutations, the length of the bar graph represents the frequency of mutations with the target gene. (A) The concurrent mutation of *KDM5A* with *MUC16, TP53, SRSF2*, and *NRAS* in s‐AML. (B) The concurrent mutation of *MUC16* in s‐AML, with *KDM5A* and TP53 was more prominent. (C) All patients with *MUC16* mutation in AML co‐occurred with *CEBPA* mutation. (D) The concurrent mutation of *MUC16* in MDS, MPN, and MDS/MPN.

*KDM5A* and *MUC16* mutations were present at low frequencies in MN, but with a higher frequency in s‐AML compared to other subgroups. *KDM5A* mutation was only identified in two patients with s‐AML (8.69%, 2/23), and always co‐occurred with *MUC16* and *TP53* mutations (100% 2/2), and co‐occurred with *SRSF2* and *NRAS* in 50% (1/2) patients (Figure 9A). *MUC16* mutations occurred in three patients with s‐AML (13.04%, 3/23), along with *KDM5A* and *TP53* mutations (66.7% 2/3). Other concurrent mutations included *DNMT3A, FLT3, SRSF2*, and *NRAS* (Figure 9B). However, *MUC16* mutations were found in four de novo AML patients (2.81%, 4/142), and co‐occurred with *CEBPA* in all of the patients. Other concurrent mutations included *NRAS, NOTCH1, DNMT3A, GATA2, NF1*, and *WT1* (Figure 9C). In MDS and MPN, *MUC16* mutations occurred in three patients (2.31%, 3/130), but there were no particularly prominent concurrent mutations (Figure 9D).

## 4 DISCUSSION

Genetic alterations are critical in MN pathogenesis and many gene variants have been identified to guide diagnosis, prognosis, and precise treatment. Our results revealed that 88.11% of patients with MN had at least one pathogenic gene mutation detected by NGS; the sensitivity was consistent with that of a previous study.^16^ *TET2* gene mutation was the most common in MN, followed by *GATA2, ASXL1, FLT3, DNMT3A*, and *TP53*. The gene mutation structure was differed slightly from a recent study; the study demonstrated *ASXL1, SRSF2*, and *TET2* mutations were more common.^17^ *NPM1, DNMT3A, FLT3*, and *NRAS* had a high mutation frequency in AML.^6^, ^18^ In this study, epigenetic abnormalities were the most prominent in ML, where *TET2, ASXL1, GATA2*, and *FLT3* had the highest mutation frequency (≥20%), followed by *NPM1, NRAS*, and *DNMT3A* (10%–20%); the results were slightly different from those of previous studies; however, these genes are common in AML.

We found that older patients with ML had shorter survival time in the final Cox regression model. Further analysis showed a higher incidence of genetic mutation events in the older patient group, which could explain the lower OS. Previous epidemiological surveys on AML also found that older patients had poorer OS than younger patients,^19^, ^20^, ^21^ gene mutations of myeloid transcription factor were prominent in younger patients, and epigenetic mutations were prominent in older patients.^22^, ^23^ In addition, another study confirmed *RUNX1, TP53, IDH 2*, and *SF3B1* gene mutations and epigenetic genes were more frequent in older patients.^24^ TET2 is considered an age‐associated mutation that contributes to myeloid expansion.^25^

In addition, the intermediate group had superior OS compared to the good and poor karyotype groups, which demonstrated the diversity and complexity of the prognostic factors in ML. This may be related to the different treatment methods used in different karyotype groups. Patients in the intermediate group received more allogeneic HSCT than those in the good group, which improved the overall survival; in the good group, autologous HSCT or constant chemotherapy was recognized as an appropriate treatment. This indicates that allogeneic HSCT is an effective method to improve the OS of patients with ML. Unfortunately, genetic mutations have no significant prognostic significance compared to the wild type in ML. However, some studies have demonstrated different results; *ASXL1, RUNX1, TP53*, and *FLT3‐ITD* mutations are associated with poor prognosis,^21^ and *DNMT3A, IDH1*, and *IDH2* mutations have not been clearly defined as prognostic factors.^9^ *TET2* and *GATA2* are commonly mutated genes in MN; however, the value of *TET2* was not accordant. In AML studies, patients with *TET2* mutations may be more sensitive to cytotoxic therapy; however, *TET2* is not an independent prognostic marker.^26^

Regarding the gene mutation landscape of MDS, a recent study reported that *TET2, SF3B1, ASXL1, SRSF2*, and *RUNX1* were highly mutated in MDS patients,^27^ which is consistent with our study. In MPN and MDS/MPN, *ASXL1* mutations appeared with the highest frequency, whereas *JAK2, CARL*, and *MPL* mutations were present at low frequencies, similar to previous studies.^28^ Another study investigating 426 MDS patients showed that *CBL, IDH2, ASXL1, DNMT3A*, and *TP53* were associated with shorter OS and poor prognosis.^29^ *SRSF2* and *RUNX1* mutations were associated with reduced survival in MDS^30^; however, the *SF3B1* mutation was a predictor of favorable prognosis in MDS and MDS/MPN.^31^, ^32^ *TET2* and *GATA2* mutations are controversial in MDS.^33^ Clinical features seemed to have no impact on prognosis in patients with MDS, MPN, and MDS/MPN. However, patients with the *BCORL1* mutation had a significantly reduced OS (*p* = 0.04); *BCORL1* (*BCL6* corepressor‐like1) gene is a transcriptional corepressor that helps inhibit E‐cadherin.^34^ *BCORL1* mutation is a low‐frequency mutation associated with a poor prognosis in MDS and an incidence of AML transformation as reported previously.^35^, ^36^

*FLT3, TP53, SRSF2, MUC16*, and *KDM5A* mutations were more frequent in s‐AML than in MDS and MPN. Furthermore,*TP53, U2AF1, SRSF2*, and *KDM5A* mutations were significantly more frequent in s‐AML than in ML. These differential gene variants may be mutations that promote disease progression. According to previous reports, NGS detection revealed that *TP53, SRSF2*, and *TET2* mutations were poor prognostic factors; especially *SRSF2* mutations, which could accelerate the transformation of MPN to AML.^37^ Moreover, in an MDS study, the results indicated that *NRAS, KRAS, PTPN11*, and *FLT3* mutations promote the transformation of MDS, while *NPM1, WT1*, and *IDH2* mutations were common in MDS‐transformed s‐AML.^38^ *TP53, RUNX1, ETV6, EZH2*, and *ASXL1* are high‐risk genes that promote the transformation of MDS. *ASXL1, EZH2*, and *SRSF2* mutations were associated with poor prognosis in primary myelofibrosis (PMF), and the patients could easily transform to s‐AML.^39^ *U2AF1* is a recurrent somatic mutation in the splicing factor, and at a low frequency in AML and MDS, *U2AF1* can activate immune pathways and affect myeloid malignancies.^40^, ^41^ Most studies have shown that patients with *U2AF1* mutations were associated with poor survival in MDS and AML, but this was still controversial.^39^, ^42^, ^43^, ^44^

*KDM5A* is a low‐frequency gene and was observed in two patients with s‐AML. It is a pathogenic gene and a marker for efficacy response in a variety of tumors.^45^ A study of acute promyelocytic leukemia (APL) indicated that KDM5A contributes to the blockage of cell differentiation.^46^ Similarly, a recent study suggested that KDM5A is associated with cell apoptosis in AML cell lines and may be a potential target for demethylation therapy in AML.^47^ However, the clinical value of s‐AML has rarely been reported. *MUC16* (also known as CA‐125) plays an important role in tumorigenesis, proliferation, migration, and invasion and is an important target for the diagnosis and treatment of gynecological tumors.^48^, ^49^ Both in vitro and in vivo studies have proven that this marker may be a potential approach for treating hard‐to‐cure AML.^49^ It was interesting to find concurrent mutations *KDM5A* and *MUC16* in s‐AML; however, all *MUC16* mutations co‐occurred with *CEBPA* in AML. The prominent concurrent mutation differences between diseases may be related to different pathogeneses, and future studies are needed to confirm this hypothesis. The limitation of this study is the limited number of cases in the s‐AML and MPN subgroups; therefore, it is challenging to fully clarify the role of gene variants in the pathogenesis and prognosis of MN.

In summary, this study provides a detailed analysis of gene profiles in MN subgroups and highlights the prognostic relevance and value of these genetic variants. These results provide evidence for further research on the function of genes in the clonal evolution of MN. Future studies are needed to dynamically monitor the changes in gene mutations at different disease stages using NGS and confirm the hypothesis that differentially expressed genes may be involved in the development of s‐AML.

## AUTHOR CONTRIBUTIONS

**Junnan Li:** Data curation (equal); formal analysis (lead); investigation (equal); methodology (lead); software (lead); validation (equal); visualization (equal); writing – original draft (lead). **Li Pei:** Project administration (equal); resources (equal); writing – review and editing (equal). **Simin Liang:** Conceptualization (equal); data curation (equal); investigation (equal); software (equal). **Shuangnian Xu:** Project administration (equal); resources (equal). **Yi Wang:** Project administration (equal); resources (equal). **Xiao Wang:** Project administration (equal); resources (equal). **Yi Liao:** Project administration (equal); resources (equal). **Qian Zhan:** Data curation (equal); resources (equal). **Wei Cheng:** Resources (equal). **Zesong Yang:** Resources (equal). **Xiaoqiong Tang:** Resources (equal). **Hongbin Zhang:** Resources (equal). **Qing Xiao:** Project administration (equal). **Jianbin Chen:** Project administration (equal); supervision (equal). **Lin Liu:** Project administration (equal); supervision (equal). **Li Wang:** Conceptualization (lead); data curation (equal); formal analysis (equal); funding acquisition (lead); investigation (equal); methodology (equal); project administration (lead); resources (equal); supervision (lead); visualization (equal); writing – review and editing (lead).

## FUNDING INFORMATION

This work was supported by the Natural Science Foundation Project of Chongqing (cstc2018jcyjAX0688, Supported by Natural Science), the Science and Health joint project of Chongqing (2018ZDXM001), and the Education Commission Foundation of Chongqing (KJ1702017), Key Projects of Science and Technology Commission in Yuzhong District of Chongqing(20190121).

## CONFLICT OF INTEREST STATEMENT

The authors declare that they have no conflict of interest.

## ETHICS STATEMENT

This study was approved according to the ethical guidelines of the First Affiliated Hospital of Chongqing Medical University (2021‐342) and was performed according to the Declaration of Helsinki. Written informed consent was obtained from the patients.

## Data Availability

The datasets generated and analyzed during the current study are publicly available from the corresponding author upon reasonable request.
